# From Seeing to Simulating: A Survey of Imaging Techniques and Spatially-Resolved Data for Developing Multiscale Computational Models of Liver Regeneration

**DOI:** 10.3389/fsysb.2022.917191

**Published:** 2022-06-06

**Authors:** Aalap Verma, Alexandra Manchel, Justin Melunis, Jan G. Hengstler, Rajanikanth Vadigepalli

**Affiliations:** 1 Daniel Baugh Institute for Functional Genomics and Computational Biology, Department of Pathology, Anatomy, and Cell Biology, Thomas Jefferson University, Philadelphia, PA, United States; 2 IfADo-Leibniz Research Centre for Working Environment and Human Factors, Technical University Dortmund, Dortmund, Germany

**Keywords:** liver regeneration, multiscale modeling, noninvasive imaging, liver physiology, microscopy

## Abstract

Liver regeneration, which leads to the re-establishment of organ mass, follows a specifically organized set of biological processes acting on various time and length scales. Computational models of liver regeneration largely focused on incorporating molecular and signaling detail have been developed by multiple research groups in the recent years. These modeling efforts have supported a synthesis of disparate experimental results at the molecular scale. Incorporation of tissue and organ scale data using noninvasive imaging methods can extend these computational models towards a comprehensive accounting of multiscale dynamics of liver regeneration. For instance, microscopy-based imaging methods provide detailed histological information at the tissue and cellular scales. Noninvasive imaging methods such as ultrasound, computed tomography and magnetic resonance imaging provide morphological and physiological features including volumetric measures over time. In this review, we discuss multiple imaging modalities capable of informing computational models of liver regeneration at the organ-, tissue- and cellular level. Additionally, we discuss available software and algorithms, which aid in the analysis and integration of imaging data into computational models. Such models can be generated or tuned for an individual patient with liver disease. Progress towards integrated multiscale models of liver regeneration can aid in prognostic tool development for treating liver disease.

## 1 INTRODUCTION

The ability of the mammalian liver to regenerate lost tissue mass following injury has been a topic of investigation for decades, but has recently come into focus as a tool for clinical intervention in hepatocellular carcinoma and live liver transplant patients (Poon et al., 2004; Stoot et al., 2013). Liver regeneration is a coordinated, multiscale phenomenon involving a complex interaction network between intracellular and systemic-level mechanisms (Taub, 2004; Fausto et al., 2006; Michalopoulos, 2014; Michalopoulos, 2017). Systemically, there is an increase in portal blood pressure seen immediately following liver resection or injury, while at the tissue level, there is active remodeling of the extracellular matrix, and at cellular and subcellular levels, there is non-parenchymal cell recruitment via initiation of cell-intrinsic signaling. Disruption of this coordinated sequence of processes, which can last several days across multiple spatial scales, may lead to deficits in liver mass recovery, as clinically observed in chronic liver disease (Orrego et al., 1981; Koteish et al., 2002).

Despite decades of study, our understanding of the regulatory mechanisms underlying liver regeneration and repair, remains incomplete. This lack of understanding limits our ability to intervene in cases where a resected liver fails to regenerate to a level required for supporting normal physiological function. Therefore, it remains a challenge to predict the outcome of patient surgical resection or liver transplant. By leveraging large data collection and integration methods with incredible ease, as a result of the “-omics” revolution, computational modeling can be utilized to potentially identify and quantify key control points within the extensive molecular interaction network. Such modeling techniques will play an essential role in bridging the gap between clinical assessment of patients and prediction of surgical intervention viability as it pertains to liver disease.

Development of computational models capable of successfully predicting liver regeneration and providing mechanistic insights into regenerative phenomena, requires integration of information spanning different spatial and temporal scales. For example, although phenomenological models can be developed relying solely on longitudinal liver volumetric data, it will have limited practical applicability. To gain predictive power, liver regeneration models should translate molecular pathways to intercellular interactions, matrix remodeling, and ultimately liver mass recovery. In addition, incorporating information at smaller spatial scales is of particular importance in the liver as its functional capabilities are directly linked with its morphology at the tissue level. For instance, functional heterogeneity of hepatocytes within different lobular locations, termed liver zonation, is a well-studied phenomenon (Jungermann and Kietzmann, 1996; Braeuning et al., 2006; Gebhardt and Matz-Soja, 2014). Hepatocyte function and regeneration are strongly regulated by localized interactions with non-parenchymal liver cell types. Dynamically tracking and deciphering zonal preferences of cellular activity, cell neighborhoods, and cell-microenvironment interactions, could form the basis of building multiscale computational models of liver regeneration with strong predictive capabilities.

In this work, we review commonly employed imaging methods utilized for visualization of liver histology and functionality at various length scales, that are capable of aiding multiscale model development of liver regeneration. Additionally, we discuss the software applications available for image analyses. Finally, we provide brief overviews of widely available computational models of liver regeneration and function, based on the select techniques discussed, and provide future perspectives on multiscale modeling of liver regeneration. Molecular data obtained from single cell omics platforms have informed and advanced the current understanding of liver tissue function. Furthermore, integration of such data into computational models have improved their parameterization and predictive power. However, in this review we focus on imaging-based modalities and how they can inform multiscale models at the organ-, tissue- and cellular-level.

## 2 ORGAN-LEVEL IMAGING AND MODELING

**FIGURE 1 |:**
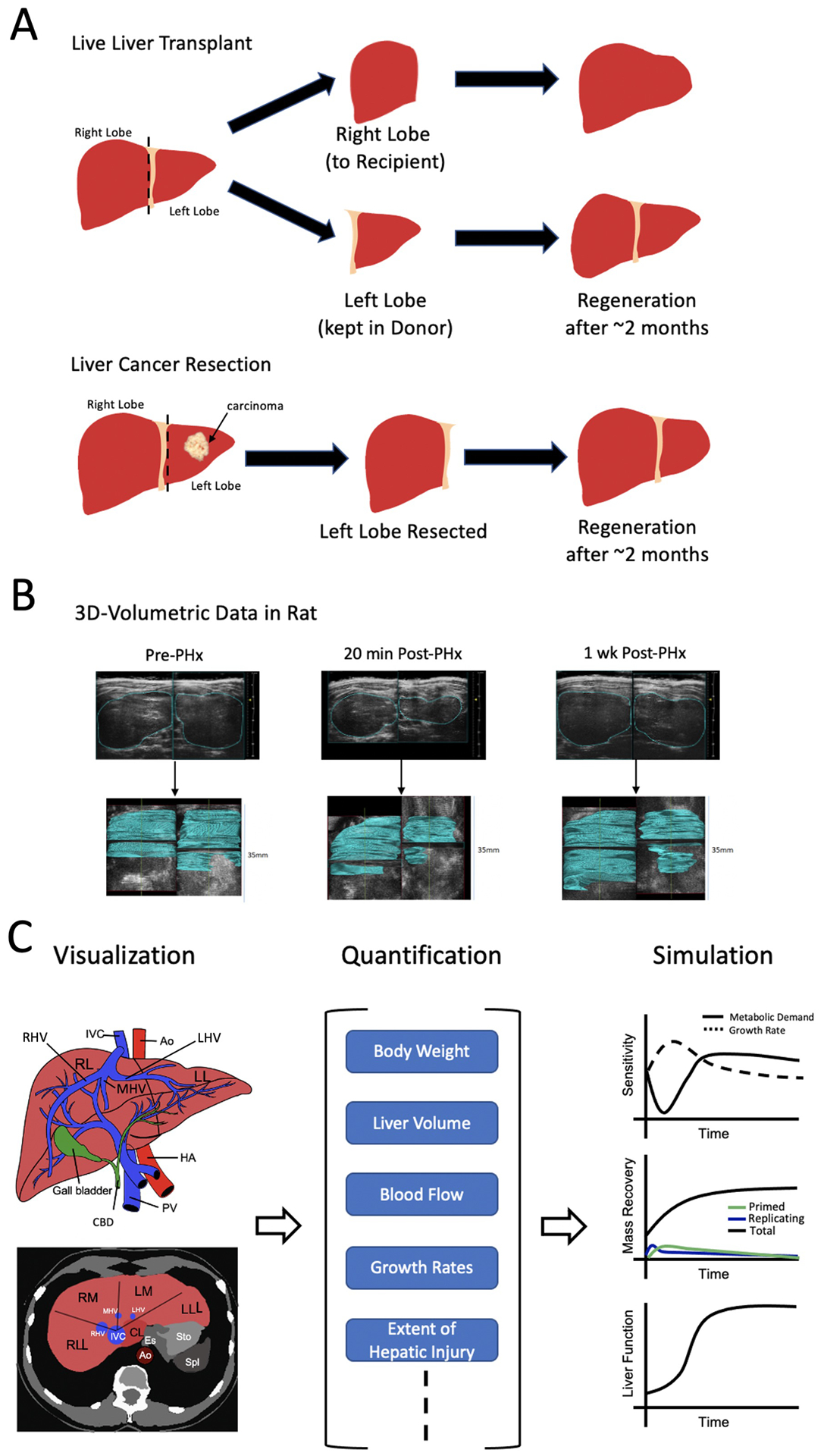
Organ-Level Approach. **(A)** Two possible scenarios leading to liver regeneration. (Top) Live liver transplant where the right lobe is resected and given to the recipient while the left lobe is kept intact in the donor. In either case, regeneration of the liver takes roughly 2 months. (Bottom) Following identification of carcinoma in the left lobe of the liver, it is resected. Again, after about 2 months the liver has regenerated. **(B)** 3D ultrasound images prior to and following partial hepatectomy (PHx) in a rat model are shown. After 1 week, the liver has nearly completed the regeneration process. At each time point, stacking of ultrasound images (shown in blue) allows for full 3D reconstruction. **(C)** Visualization of the liver at the organ-level (left) can be quantified (middle) leading to the possibility for simulation (right). At the organ-level, only general volumetric measurements can be made such as body weight, total volume, blood flow, etc,. Therefore, modeling and simulation running can only be conducted for broad concepts such as hepatic growth rate vs metabolic demand, mass recovery and general liver function. IVC = inferior vena cava, Ao = Aorta, HA = hepatic artery, PV = portal vein, CBD = common bile duct, RL = right lobe of liver, LL = left lobe of liver, RLL = right lateral lobe of liver, RM = right medial lobe of liver, LM = left medial lobe of liver, LLL = left lateral lobe of liver, CL = caudate lobe, RHV = right hepatic vein, MHV = medial hepatic vein, LHV = left hepatic vein. Sources: Cook et al., 2015; Cook et al., 2018. Figure made with BioRender.

Non-invasive measurement of liver volume during regeneration provides key temporal data, necessary for development of computational models. Imaging platforms capable of visualizing whole liver samples with high enough resolution for organ morphology detection, find important clinical applications. A vital aspect of improving outcomes of surgical liver resection in cancer patients and live liver donors, is surgical planning. The liver’s regenerative potential following cancer resection or live liver transplant, is shown in Figure 1A. Surgical planning seeks to achieve the precise balance of maximal target lesion removal, maximal sparing of functional liver remnant volume, and minimal surgical invasiveness (Wang et al., 2017). Therefore, it requires accurate knowledge of important hepatic structure location, including that of major hepatic blood vessels and bile ducts. A 3D visualization and virtual reconstruction of the entire organ can provide such information (Figure 1B). Numerous techniques have been developed in this regard. Here, we discuss three broad classes of whole liver imaging–computed tomography (CT), magnetic resonance imaging (MRI), and ultrasound.

CT is based upon differential absorption of x-rays by water, bones, and soft tissue. Contrast differences appear on CT detectors when components of differential absorbance are present in the image, thus yielding information about tissue morphology. Next, MRI relies on the measurement of time-variant magnetic spins of hydrogen atoms under constant and dynamic magnetic fields to identify different structures and components within the scanned area. Finally, ultrasound uses acoustic impedance, i.e., the ability of different materials to reflect sound, and echoes to map the depth as well as composition of structures within a tissue. While all three techniques can be utilized for volumetric measurements of the liver, MRI offers higher soft tissue contrast compared to CT and ultrasound. A recent review article highlights the application of MRI-based liver modeling in understanding liver biomechanics and improving liver disease treatment (Seyedpour et al., 2021). Studying biomechanics of the liver aided by MRI allows for predictive modeling of liver diseases to identify and better understand the progressive stages ahead. Temporal and spatial data such as blood volume, blood velocity, and wall shear stress (WSS) can be extracted from images to make such determinations of disease state. Reconstruction of acquired whole liver images remains a challenging problem. Nevertheless, with the increase in computational power available at the disposal of researchers and technicians, increasingly sophisticated algorithms have become available for this purpose. Liver segmentation algorithms employed for partitioning of the liver into parenchyma and non-parenchyma, are commonly classified into two major categories–semi-automated segmentation and automated segmentation. Descriptions and comparisons of these algorithms can be found in reviews Luo et al., 2014 and Mharib et al., 2012. Besides proprietary software available for analysis of whole liver images, such as Synapse Vincent (Fujifilm^®^, Ohshima, 2014) and Osirix (Tzourio-Mazoyer et al., 2002), several open-source options exist for research and/or surgical purposes. Applications such as Itk-Snap (Yushkevich et al., 2006), 3D Slicer (Fedorov et al., 2012), and VR-Render (D’Agostino et al., 2013), are capable of segmenting DICOM (Digital Imaging and Communications in Medicine) formatted images obtained from any of the three organ-level imaging techniques: CT, MRI or ultrasound (see Strakos et al., 2015 for brief review of some of the listed applications). For images acquired by CT, pipelines have been developed to reconstruct whole livers using generic applications, such as ImageJ (Dello et al., 2007) and Adobe^®^ Photoshop (Lu et al., 2004). A recent study introduced a new segmentation method for analyzing images acquired by CT, namely 3D U-Net, which utilized a convolution neural network in addition with position features such as the spine, body surface, and sagittal plane, to improve the accuracy of liver segmentation (Jiřík et al., 2021).

MRI, CT, and ultrasound seek widespread clinical applications in diagnosis of focal and diffused liver conditions, including steatosis, hepatocellular carcinoma, colorectal liver metastases, and liver fibrosis. Apart from diagnostic and surgical planning applications, whole liver imaging provides essential information for modeling liver regeneration–liver volume time series data. Volumetric and temporal monitoring of a liver during regeneration serve as simple and basic metrics necessary for fitting a dynamic liver regeneration model. Yamamoto et al. (2016) demonstrated the clinical application of CT imaging and computational modeling in predicting patient-specific liver regeneration profiles. The authors used a combination of CT imaging data and preclinical data such as BMI, blood loss, resected volume, etc,. from 123 patients to identify key perioperative measures capable of discriminating between cases of normal and suppressed regeneration. They then developed a phenomenological computational model which was validated in a 39-patient cohort. This type of modeling provides practical application in clinical settings, as patients who need close follow-up attention after liver resection can be identified by the model. A similar model development workflow to that of Yamamoto et al. (2016) can be observed in Figure 1C.

Owing to a lack of spatial and mechanistic detail, ODE-based computational modeling of liver regeneration dynamics at the organ level are most prominent. A variety of commercial (e.g., Matlab) and open source (e.g., R, Python) languages/applications are available for this purpose. Recent efforts have seen an increase in freely accessible computational models with a particular focus on model sharing to encourage use of predictive and dynamic modeling approaches in research and medicine. Databases, such as BioModels (Li et al., 2010), and standardized, open-source formats, such as Systems Biology Markup Language (SBML) (Hucka et al., 2003), have gained prominence, as they aid in the ease of model sharing. Numerous applications exist for the reuse of SMBL models; one application able to perform interactive parameter scans, with only the click of a few buttons is COPASI (Mendes et al., 2009).

## 3 LOBULE-LEVEL IMAGING AND MODELING

**FIGURE 2 |:**
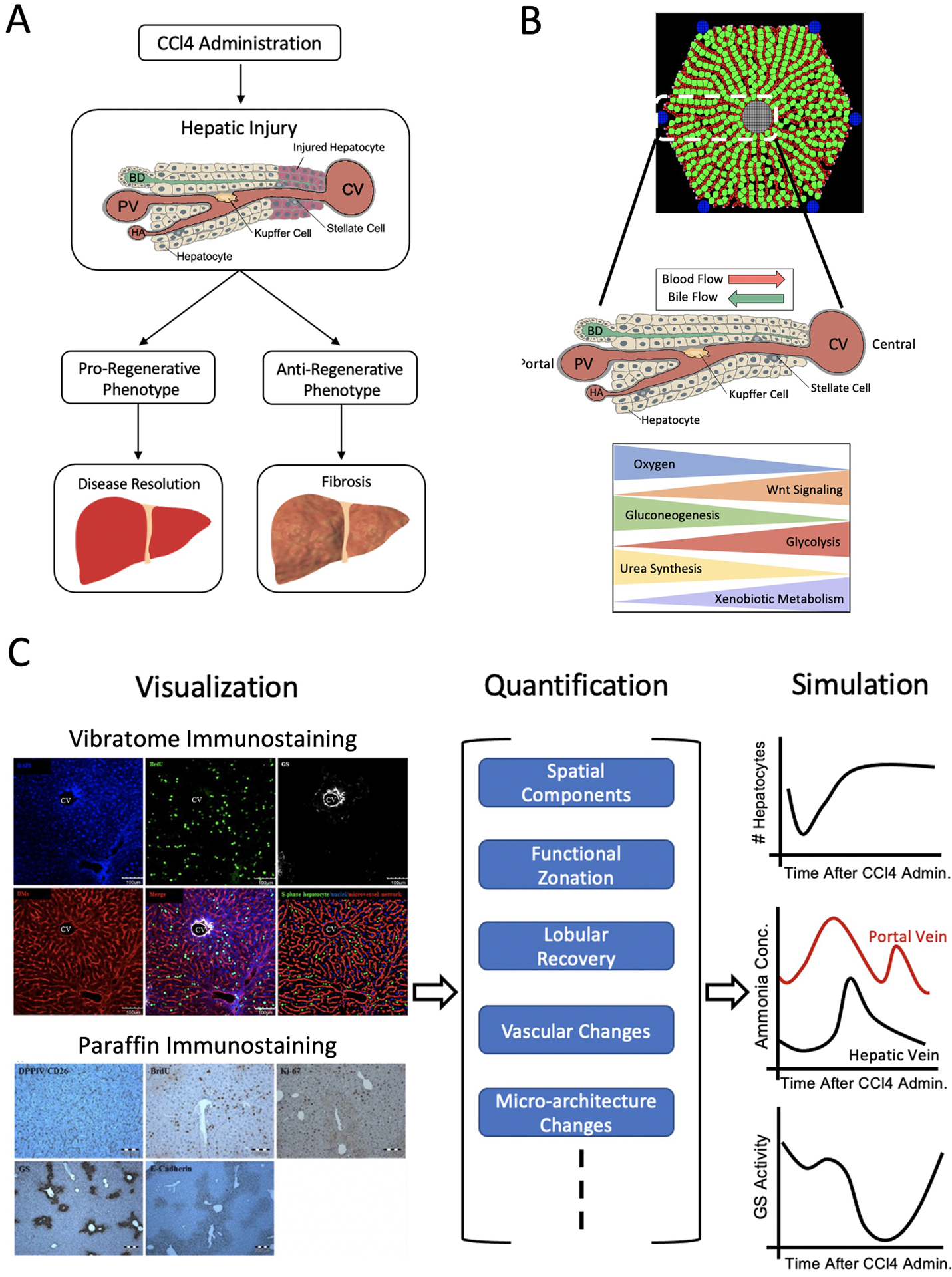
Lobule-Level Approach. **(A)** Upon regional injury induction, for instance by CCl4 administration, which induces pericentrally-located damage, hepatocytes undergo either a pro-regenerative or anti-regenerative phenotype. If they exhibit a pro-regenerative phenotype, the damage can be resolved whereas if they exhibit an anti-regenerative phenotype, further damage may occur, leading to fibrosis. **(B)** The liver lobule is characterized by the formation of hepatocytes in a string-like fashion along the porto-central axis. Blood flows through the sinusoids (red) from the portal vein (PV) and hepatic artery (HA) to central vein (CV), while bile flows through bile canaliculi (green) from the central area to the portal bile duct (BD). There exists a zonated set of processes, such as urea synthesis and xenobiotic metabolism, with gradient-like behavior along this axis. **(C)** Visualization of the lobule through vibratome or paraffin staining (left) can be used as a means for quantification of spatial components, functional zonation, lobular recovery, etc,. (middle). Such information allows for simulation (right) of changes in lobular hepatocyte, metabolite concentrations and zonal activity. Markers use for vibratome and/or paraffin staining: DAPI for S-phase negative hepatocytes, BrdU for S-phase positive hepatocytes, GS for pericentral hepatocytes, DMs for hepatic sinusoids, DPPIV/CD26 for bile canaliculi, Ki67 for proliferating hepatocytes, E-cadherin for basolateral membranes of periportal hepatocytes. Sources: Hammad et al., 2014; Birchmeier, 2016; Ghallab et al., 2016. Figure made with BioRender.

Although whole organ imaging can provide liver volumetric data through the various stages of liver regeneration, it cannot yield information on liver repair dynamics that operate at smaller spatial scales. For instance, preferential pericentral necrosis, due to CCl4- (Yu et al., 2002) or acetaminophen- (Black, 1984) induced toxicity, leaves the organ-level structure unchanged despite inducing mechanisms of tissue recovery, similar to that of liver regeneration (Fausto, 2004). However, if the organ is unable to regenerate tissue mass, thereby ameliorating the induced toxicity, fibrosis may occur (Figure 2A). The mechanisms behind preferential liver injury, as well as repair, are at least partly guided by liver zonation. Zonation of the hepatocyte molecular profiles within liver lobules leads to an overarching spatio-temporal organization of liver regeneration and repair processes, the understanding of which is crucial for building accurate models of liver regeneration (Figure 2B). Therefore, elucidating and visualizing processes at smaller spatial scales is necessary for integration of spatio-temporal aspects into liver regeneration modeling.

The techniques predominantly employed for imaging mechanisms involved in liver regeneration and repair at the lobular scale are bright field and confocal microscopy. The use of bright field microscopy in imaging regenerating livers relies on the incorporation of BrdU (bromodeoxyuridine), an analog of thymidine, into newly synthesized DNA (de Graaf et al., 2011). This becomes specifically relevant when injured hepatocytes are spatially localized within liver lobules, as is the case of CCl4-induced injury (Yu et al., 2002; Hoehme et al., 2010). Use of BrdU staining can provide temporally resolved information regarding variations in proportion and localization of replicating hepatocytes (Soames et al., 1994). Alternatively, confocal imaging has been extensively used for imaging and characterization of liver regeneration at the lobular level (Hammad et al., 2014). Confocal microscopy provides the additional advantage of imaging at varying tissue depths.

Confocal imaging enables quantification of liver regeneration dynamics as well as cell-type specific resolution through the use of fluorophores, such as dyes and quantum dots, and fluorescent transgenic animal models. Furthermore, confocal imaging with cell-type resolution can provide information beyond what has already been discussed. For instance, it has been observed with multiplexed confocal imaging that hepatocyte repopulation occurs along liver microvessels after necrotic lesion formation in the pericentral lobular region (space surrounding the central vein) resulting from preferential hepatocyte death due to CCl4 administration (Hoehme et al., 2010). Hoehme et al. (2010) developed a quantitative mathematical model using measured 3D changes in liver structure prior to and following CCl4 damage predicting a previously unrecognized mechanism, which is essential for liver regeneration. Specifically, during regeneration daughter hepatocytes align along the orientation of the closest sinusoid, a process which they termed “hepatocyte-sinusoid alignment”.

At the lobular level, topological characterization and quantitative analysis of imaged lobules plays a vital role in identifying key spatio-temporal aspects of tissue repair. Several open-source options are available for analysis of images acquired through bright field and confocal microscopy techniques. The most popular among these are Cell Profiler (Kamentsky et al., 2011) and ImageJ (Schneider et al., 2012). Both applications include a wide array of powerful techniques and algorithms for image analysis, such as automated segmentation, manual segmentation, thresholding, object tracking, and 3D reconstruction and rendering functionalities. Vital to liver regeneration is the proliferation of cells. LEVER is an open-source tool that relies on minimal human validation not only to segment and track cells, but also to lineage proliferating cells, thereby allowing analysis of behavioral differences across generations and between different lineage branches and trees (Winter et al., 2016). This tool provides the unique ability to understand and quantify the patterns of proliferating liver cells during both development and regeneration. TiQuant (Friebel et al., 2015), a specialized tool for quantitative analysis and reconstruction of liver imaging data, is of special note. Provided a confocal image stack with multiplexed immunostaining of hepatocytes, hepatocyte nuclei, and bile canalicular/sinusoidal endothelial cells, TiQuant is capable of providing a wealth of quantitative and topological information such as microvessel dimensions and volumes, hepatocyte volumes, cell-cell contacts, etc., in addition to a 3D rendering of the imaged volume.

Inclusion of spatial context and functional zonation at the lobular scale, as depicted in Figure 2C, while adding crucial detail, also introduces additional complexity in computational modeling of liver function and regeneration. Agent-based modeling is a specific type of modeling strategy capable of capturing the spatio-temporal complexity at the lobular scale. In agent-based modeling, the system is modeled as a collection of agents, which are able to individually assess and make decisions about a given scenario (Bonabeau, 2002). Lobular scale computational models have previously been developed to simulate and investigate zonated metabolism of ammonia and xenobiotics in-silico (Ohno et al., 2008; Sheikh-Bahaei et al., 2010; Diaz Ochoa et al., 2012; Fu et al., 2018; Griffin and Bradshaw, 2019). More specifically, Fu et al. (2018) built a virtual hepatic liver model of xenobiotic transport and metabolism to investigate regional variations in microdosimetry. The authors found that persistent simulations, which were characterized by a constant xenobiotic input, in combination with varying transport and metabolism parameters showed one of following hepatic steady-state patterns: lobular-wise uniform, preferentially peripheral (radially varying) or preferentially periportal (both radially and azimuthally varying).

Specific to liver regeneration, a cell-oriented agent-based modeling approach has yielded successful results in capturing spatio-temporal dynamics of tissue recovery at the lobular scale, as described by Hoehme et al. (2010). Although the model relies exclusively on elastic properties of cells and forces rather than biochemical aspects, it was successful in predicting hepatocyte alignment during liver regeneration. Agent based modeling strategies have also been expanded to include disease context by studying growth of fibrotic lesions induced by CCl4 administration, as shown in Dutta-Moscato et al. (2014). In the Dutta-Moscato et al. (2014) paper, the agent-based model described both molecular and histopathological aspects of inflammation and fibrosis in a CCl4-injured liver. The model was capable of recapitulating key histopathological and macroscopic properties of CCl4-injured livers, including increased liver stiffness, collagen deposition, and disruption of the regular lobular structure.

Although models of liver regeneration in a 2D or 3D spatial context can be built in regular lattice geometries using various programming languages with strong numerical capabilities, specialized applications exist for the purpose of user ease. Modeling of CCl4 induced fibrosis progression in 2D is detailed in Dutta-Moscato et al. (2014) and was performed using SPARK (Simple Platform for Agent-based Representation of Knowledge), an agent-based modeling framework designed for systems-level biomedical model development (Solovyev et al., 2010). Other applications allow for incorporation of tissue-level morphology into the model building process, which serves as a computational improvement from regular lattice modeling. The modeling work of Hoehme et al. (2010), which utilizes CellSys (Hoehme and Drasdo, 2010), a modular software for simulating the growth and organization processes in multi-cellular systems, includes detailed spatial structures of liver vasculature acquired using confocal microscopy. An open-source application capable of modeling spatial structure, CompuCell3D (CC3D; Swat et al., 2012), provides functionalities for inclusion of tissue morphology and integration of published and curated SBML models. Additionally, CompuCell3D comes equipped with specialized plugins for cell death and mitosis, chemical field gradients, cell secretion, etc,. Sluka et al. (2016) utilized CompuCell3D to build a multi cell sinusoid tissue model of xenobiotic metabolism in which flowing blood and diffusive and active transport processes were implemented utilizing CC3D’s specialized plugins. A combination of both tissue level morphological and functional details, along with easy-to-use computational applications, can provide a means for inclusion of spatially dependent mechanistic details for in-silico liver regeneration modeling.

## 4 CELLULAR-LEVEL IMAGING AND MODELING

**FIGURE 3 |:**
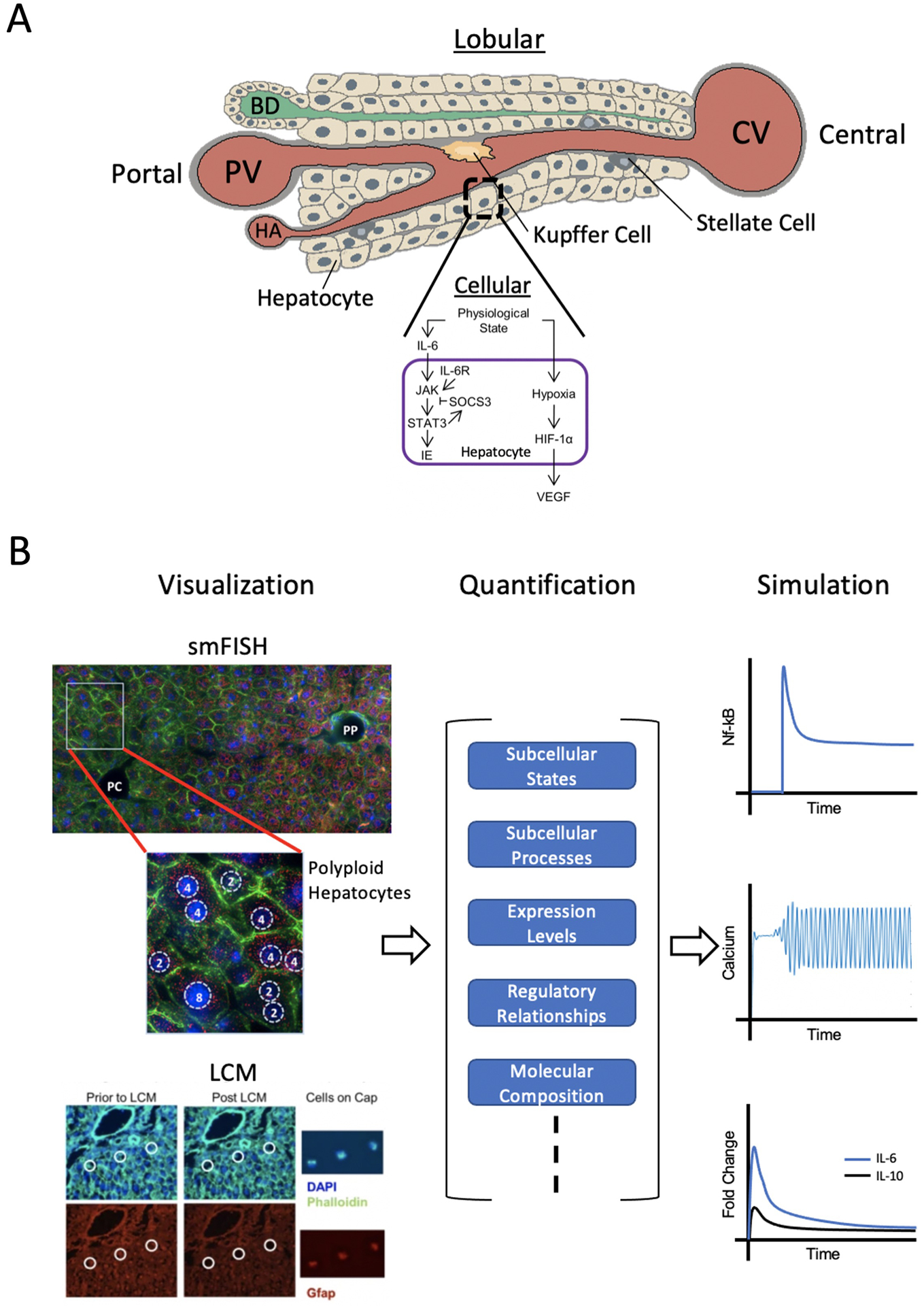
Cellular-Level Approach. **(A)** Individual hepatocytes within the liver lobule each have their own set of subcellular processes and functions. The cellular signaling network within hepatocytes during regeneration is shown. **(B)** Single cell visualization and analysis (left) by smFISH (single molecule fluorescence *in situ* hybridization) or LCM (laser capture microdissection) can provide quantitative information (middle) on subcellular states and processes as well as regulatory relationships, etc,. Simulations using this data can be conducted (right) to model species such as hepatic NF-kB, calcium spiking and cytokine dynamics during regeneration. smFISH: red = single mRNA molecules of Pck1 (marker of gluconeogenesis), blue = DAPI-stained nuclei, green = phalloidin membrane staining, PP = periportal, PC = pericentral. Polyploid hepatocytes have one or two nuclei, each with either two, four, or eight copies of each chromosome. LCM: blue = DAPI-stained nuclei, green = Phalloidin staining for cell boundaries, red = Gfap staining for hepatic stellate cells (HSC’s). Definitions: IL-6 = interleukin 6, IL-6R = IL-6 receptor, JAK = Janus kinase, STAT3 = signal transducer and activator of transcription 3, SOCS3 = suppressor of cytokine signaling 3, IE = immediate early genes, HIF-1α = hypoxia inducible factor 1α, and VEGF = vascular endothelial growth factor. Sources: Kuttippurathu et al., 2014; Halpern et al., 2015; Cook et al., 2018. Figure made with BioRender.

While computational modeling based on imaging at the organ and lobule level are essential for building predictive models of liver regeneration, they fail to elucidate subcellular states and processes that may determine the fate of the regenerating liver. Figure 3A shows only a small portion of molecular processes occurring in the hepatocyte, which contribute to the regenerative process and therefore serve as powerful predictive measures of regenerative potential at the lobular and organ level. An important aspect of liver regeneration and repair is the involvement of non-parenchymal cells in control and regulation of processes at the cellular and microenvironmental level (Malik et al., 2002; Tanaka and Miyajima, 2016). The function of nonparenchymal cells, specifically, Kupffer cells, hepatic stellate cells, and sinusoidal endothelial cells in liver function and regeneration have been well documented. The spatial location and molecular states (the intracellular mRNA and protein composition) of different cell types residing in the liver parenchyma is therefore an important consideration in liver regeneration modeling.

In the context of liver regeneration modeling, imaging at the single cell level has the potential to provide insight into the individual molecular states of hepatic cells. Characterization of molecular states across different cell types could shed light on cellular functions and interactions involved in the regulation of liver regeneration at very small spatial scales. Identification of altered molecular states and localized interactions of cells under the effect of perturbations or in the case of disease has gained considerable interest recently, as it plays a key role in shaping tissue function (Tang et al., 2010; Park et al., 2014; Patel et al., 2014). More specifically, in Park et al. (2014), the *in vivo* input type variability was analyzed in hypertensive and baseline brain tissue by high throughput qPCR. Their results indicated that there exists an organizational structure in which neuronal variability aligned with input type along a continuum of subphenotypes and corresponding gene regulatory modules. Given a physiological perturbation of hypertension, the distribution of cells within the gene expression landscape changes, however the regulatory network is altered in such a way as to produce a cellular phenotype that has adapted to the input. These results can be applied broadly to other tissues, including the liver, to further our current understanding of the relationship between cellular input and cell phenotypes. Because of the interest in molecular state characterization at the single cell level, imaging techniques have evolved to keep up with the demands. Several strategies, for instance confocal microscopy supported by smFish (single molecule fluorescent *in-situ* hybridization), can be used for characterization of molecular states of individual cells. Recent studies in the liver have demonstrated “bursty” gene expression in hepatocytes using smFish (Halpern et al., 2015) as well as zonated gene expression across entire liver lobules using a combination of smFish and single cell RNA sequencing (Halpern et al., 2017). smFISH in coordination with another single cell acquisition method, laser capture microdissection (LCM), can provide both visualization and capturing capabilities that can be used for quantification and downstream modeling and simulation (Figure 3B).

Imaging dynamic processes within the histologically complex liver parenchyma could provide further information regarding molecular trafficking and intercellular interactions under homeostatic conditions and perturbations. Such powerful technological advances in molecular imaging, enables visualization of cell-level dynamics over short time scales in live animals. While these visualization tools are not readily available in humans, animal models display incredibly comparable systems. For example, Young and Periwal (2016) showed that a mathematical model of liver regeneration originally developed by Furchtgott et al. (2009) to account for the cellular transitions and signaling processes in the rat can predict liver regeneration dynamics in five other species, including human, by scaling a single metabolic load parameter. Similar results on potential cross-species translation were shown by Cook et al. (2015) where a cellular model of liver regeneration was parameterized across rat, mouse and human scenarios by scaling the metabolic load parameter. Therefore, the opportunity now exists for use of imaging techniques in animals as a means for modeling in humans. This method of “seeing” in animals and “simulating” in humans presents a useful strategy in disease research, capable of providing many new insights, specifically to the field of liver regeneration.

An emerging imaging method capable of obtaining cell-level molecular data is MALDI (Matrix Assisted Laser Deposition Ionization) imaging mass spectrometry (Aichler and Walch, 2015). MALDI involves the application of a matrix onto tissue slices in order to extract molecules from tissue sections (Norris and Caprioli, 2013). Specific regions of the matrix, with a resolution of 5–10 μm, can then be ablated while still keeping the tissue intact and taken to downstream multiplexed mass spectrometry. MALDI allows for detection of a wide array of functionally relevant molecules such as cell intrinsic metabolites, drug metabolites, lipids, and proteins with spatial resolution on the scale of a typical cell diameter. MALDI has recently been used to analyze triglyceride content in normal and steatotic mouse livers, suggesting the use of this technique for clinical screening and estimating fatty liver (Nishikawa et al., 2014). An advantage of MALDI is its excellent mass spectrometry sensitivity, allowing for differentiation of hepatotoxic compounds, their metabolites and consequences on endogenous structures. A limitation is the still relatively low spatial resolution of approximately 20 μm, which is much higher compared to the theoretically possible 200 nm of conventional light microscopy. However, a resolution of approximately 20 μm is still sufficient for analysis of lobular zonation as the lobular radius is approximately 200 μm. Therefore, differentiation of the pericentral, midzonal, and periportal regions is still possible. For analysis of zonation, the MALDI signal can be superimposed onto immunostained tissue. This technique has been used to study the zonated distribution of acetaminophen (APAP), its glutathione adduct (GSH-APAP) and GSH levels after administration of APAP to mice (Sezgin et al., 2018). While APAP was not zonated in liver tissue, GSH-APAP and GSH depletion occurred preferentially in the cytochrome P4502E1 (CYP2E1) positive pericentral lobular region, confirming the concept that CYP2E1 metabolizes APAP to a reactive intermediate (NAPQI) that depletes GSH. Thus, MALDI is a method of choice for the label free analysis of chemicals and their metabolites in tissue.

While imaging methods, such as MALDI, are capable of extracting molecular details from tissue slices at a low resolution, ionic level imaging is only possible at a higher resolution. Such methodologies exist for dynamic quantification of ions, i.e., calcium, through *in vivo* loading of a sensitive indicator followed by high-resolution confocal microscopy. At a molecular level, calcium signaling plays a large role in the regenerative potential of the liver. Since calcium regulates many key hepatic processes such as lipid metabolism, carbohydrate metabolism, bile secretion, and choleresis, its dysregulation can lead to serious proliferative consequences. Therefore, a carefully and accurately orchestrated calcium response must prevail to cope with the stress of the injury (Oliva-Vilarnau et al., 2018). Since this response is tightly regulated, both temporally and spatially, detailing this molecular change would greatly benefit a computational model of liver regeneration. In order to gain such spatial and temporal insight into hepatic calcium content, reliable imaging techniques are necessary. As seen with many other molecular imaging methods, limited resources allow for live visualization in humans. This continues to be the case with calcium imaging as well. Therefore, rodent models have provided us with a wealth of data, capable of being applied to human systems. However, calcium imaging may produce very noisy and unreliable signals. Therefore, improvements in such imaging techniques and software analyses are essential for proper modeling, especially during liver regeneration. Recent advances in calcium imaging and modeling techniques allows for better correction, extraction and denoising of calcium signals. Two new software show great advancement in signal clarity: CellSpecks (Shah et al., 2018b), which allows for automated detection and analysis of calcium channels in live cells, and TraceSpecks (Shah et al., 2018a), which can be used for automated idealization of noisy patch-clamp and imaging data. The dual use of these software allows for calcium imaging, detection and quantification ease. Another calcium signaling software worth noting is CaSCaDe (Agarwal et al., 2017), which can be used to classify and decode calcium microdomains, thereby elucidating specific spatial patterns. Utilizing these software allows for simple incorporation of calcium signaling activity into liver regeneration or injury models.

Biochemical modeling in the liver has been performed using both ODE-based deterministic (Kholodenko et al., 1999; Verma et al., 2016) as well as stochastic (Gracheva et al., 2001) approaches. Both have been widely utilized to quantitatively describe cellular processes in single or small groups of hepatocytes. A recent study has modeled hepatocyte growth factor (HGF)-induced hepatocyte replication using an ODE-based framework; the model was able to successfully predict and validate CDK2 phosphorylation as a key control point for quiescent-to-replicating transition in hepatocytes in the absence of HGF (Mueller et al., 2015).

Incorporating cellular geometry and organelle locations in computational models could be an interesting aspect of intracellular modeling in hepatocytes. Recently, the combinatorial use of live-cell confocal and lattice light sheet spectral imaging approaches has allowed for the characterization of six organelle interactomes, providing a powerful tool for observing intercellular coordination and colocalization (Valm et al., 2017). Monte Carlo Cell (MCell, Kerr et al., 2008) is an application well suited for the incorporation of cellular geometry and organelle interactome-related information. MCell can incorporate realistic 3D spatial models of cells, while using a probabilistic approach, to model reactions within the cell as mass action kinetics. Molecular dynamics and intercellular interactions in a limited number of cells can be modeled using MCell with relative ease.

Alternative methods of imaging, such as fluorophore-based confocal and multiphoton intravital imaging, have been used to visualize the elimination of endogenous and xenobiotic molecules (Dunn and Ryan, 2017; Reif et al., 2017), study the dynamic behavior of T-cells (Benechet et al., 2017), and visualize tumor microenvironments in the liver (Tanaka et al., 2012). However, it holds the potential to provide novel insights into cellular dynamics. Specifically, cellular dynamics with spatial and temporal resolution can be elucidated during the period of non-parenchymal cell recruitment and extracellular matrix modification immediately following liver injury or resection.

## 5 TECHNICAL PERSPECTIVES: INTRAVITAL IMAGING BY TWO-PHOTON MICROSCOPY

**FIGURE 4 |:**
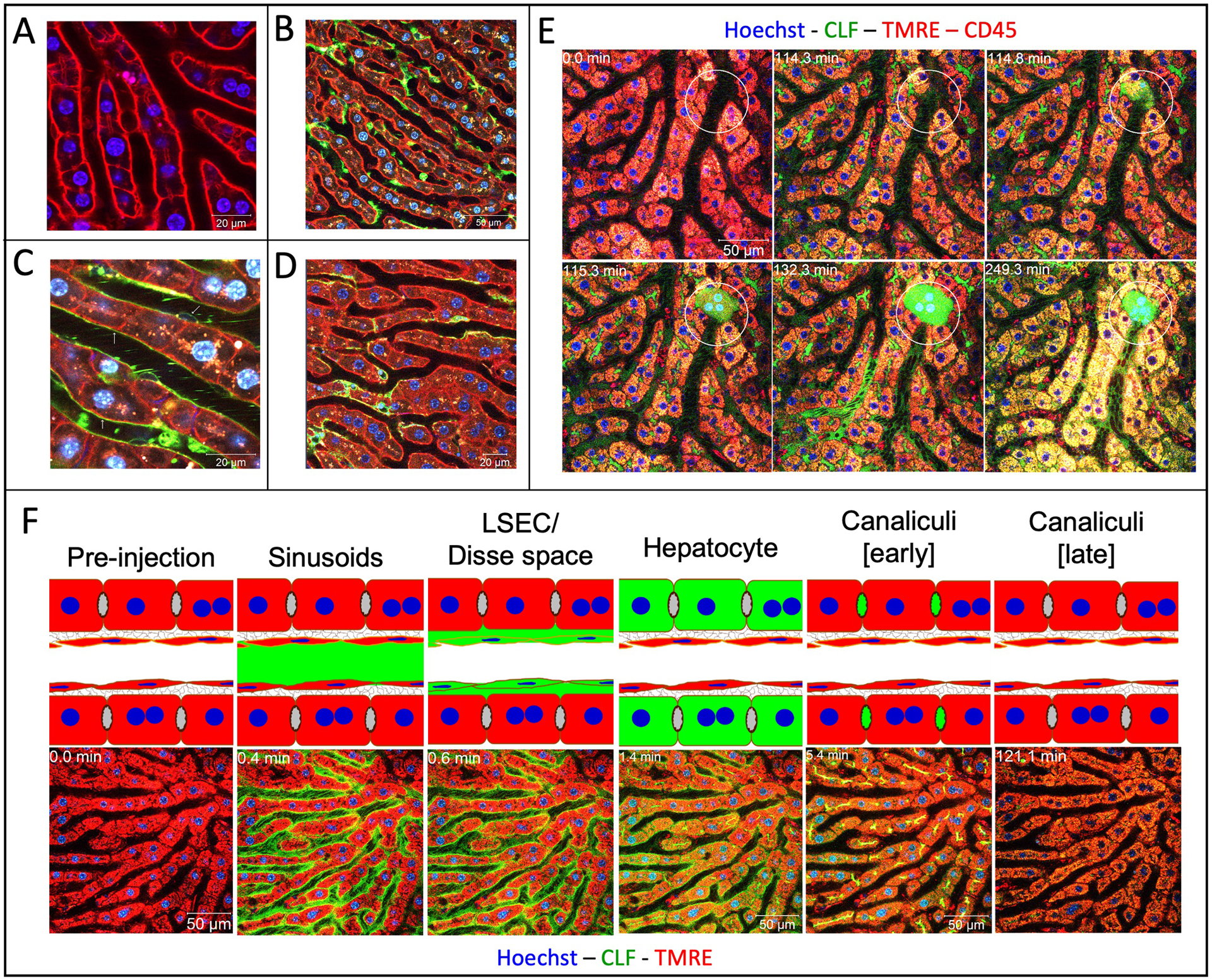
Intravital two-photon based imaging of liver cells. **(A)** mT/mG transgenic mouse showing the membranes of all cell types in red, here sheets of hepatocytes; **(B)** visualization of Kupffer cells (green) by mating to LysM-Cre mice; **(C)** visualization of sinusoidal endothelial cells (green) by mating to Tie-2-Cre mice; **(D)** visualization of stellate cells (green) by mating to Lrat-Cre mice. **(E)** visualization of the sequence of cell death events in the case of acute cholestasis induced by bile duct ligation (BDL). Indicated hepatocyte (circle) shows the following sequence of events: (1) apical membrane rupture (minute 114); (2) CLF flooding (minute 115); (3) cell death and release of CLF into the adjacent sinusoid (minute 132); and immune cell infiltration (minute 249). **(F)** visualization of bile salt transport in healthy livers. The following stations could be seen after bolus i.v. injections of CLF: (1) appearance in sinusoids; (2) enrichment in LSEC/Disse space; (3) transport to hepatocytes; and (4) secretion into bile canaliculi, which later get evacuated. Sources: Ghallab and Hengstler, 2018; Ghallab et al., 2019. Figure 4F modified from Reif et al., 2017.

Recently, it has been shown that two photon-based microscopy adds valuable information to imaging at the lobule level as well as subcellular processes (Jansen et al., 2017; Reif et al., 2017; Ghallab et al., 2019). In two-photon imaging an infrared laser hits the fluorophore with two photons simultaneously (Reif et al., 2017). Each photon transfers half of the energy required for excitation. An advantage of this procedure is that infrared lasers penetrate deeper into tissue than laser beams with higher energy. This allows for imaging of up to approximately 70 μm below the liver capsule of anesthetized mice without any phototoxicity. Further requirements of two-photon based intravital imaging are long distance objectives with high numerical aperture (>1.1), a very sensitive detection system, such as gallium arsenide phosphide (GaAsP) detectors, and an optimized inhalation anesthesia to minimize movement artefacts of the mouse. Through use of fluorescent reporter mice or intravital dyes, it is possible to visualize all relevant cell types of the liver, such as hepatocytes, sinusoidal endothelial cells, Kupffer cells and Stellate cells (Figures 4A–D; Reif et al., 2017; Ghallab et al., 2019). For analysis of liver regeneration dynamics, it is possible to take videos with fast sequences in the millisecond range. The duration of imaging is limited, however, by anesthesia, with up to 6 h of uninterrupted intravital imaging considered routine.

One advantage of two-photon based intravital imaging is that cell death events and subsequent responses can be imaged. Usually, cell death is preceded by loss of mitochondrial potential. Intravital imaging of mitochondrial potential is possible after injection of tetramethylrhodamine (TMRE) into the rodent. Mitochondria that are both negatively charged and polarized will begin to accumulate positively charged TMRE molecules. Vice versa, depolarized mitochondria accumulate less TMRE. After intoxication with APAP or CCl4, but also in liver diseases such as cholestasis, loss of TMRE-associated fluorescence usually precedes gain of propidium iodide (PI) mediated nuclear fluorescence, an intravital cell death marker. Figure 4E shows a typical scenario from a video displaying hepatocyte death caused by bile duct ligation, the initial event that leads to the so-called Charcot-Gombault necrosis or bile infarct. In these cells the mitochondrial potential decreases, followed by rupture of the apical (bile canalicular) hepatocyte membrane, and accumulation of the green fluorescent bile salt analogue cholyl-L-lysyl-fluorescein (CLF) in hepatocytes (Ghallab et al., 2019). Finally, there is neutrophil infiltration seconds to minutes later, which coincides with nuclear disintegration of the dead hepatocytes. A strength of intravital two-photon based imaging is that it helps to elucidate sequences of key events, especially if they occur within a relatively short time frame, which would otherwise be impossible with conventional histology.

One of the central functions of the liver is biliary excretion of bile acids. Xenobiotics and pharmaceutical drugs are often excreted via the biliary route. Two-photon imaging allows for the direct observation and quantification of these transport processes. The time course of transport of bile salt analogue CLF in a healthy liver is shown in Figure 4F. After bolus injection into the rodent tail vein, CLF first appears in the sinusoid, is enriched in the Dissé space and finally excreted into the bile canaliculi. After intoxication or induction of cholestasis, this transport chain is interrupted by an uptake block at the basolateral or blood side of hepatocytes. In this situation (day 21 after BDL), uptake of CLF into hepatocytes is strongly reduced. Therefore, the half-life of CLF in the circulation is much longer. This basolateral uptake block serves to prevent bile salt overloading of regenerating or cholestatic hepatocytes; it corresponds to previous reports that the basolateral export carriers MRP3, and MRP4 OSTαβ are upregulated in cholestasis (reviewed in Jansen et al., 2017). However, it should be noted that the observation of an uptake block by two-photon imaging cannot differentiate between the inability of hepatocytes to take up CLF and the ability of hepatocytes to take up CLF following immediate basolateral excretion.

## 6 LIVER VASCULATURE AND BILE FLOW NETWORK IMAGING AND MODELING

The extensive network of microvessels comprising the liver is vital to its function. The liver receives blood from the heart and gut through the hepatic artery and portal vein respectively, which is drained into the central vein after passing through sinusoids. An independent network of bile canaliculi runs throughout the organ, draining bile into the gallbladder. Any injury to the liver parenchyma perturbs the structure as well as blood flow through the liver. An increase in portal blood pressure is observed soon after liver resection. The resulting shear stress on hepatocytes could be one of the first signals instigating the downstream cohort of regenerative processes. Visualization and topological characterization of liver microvasculature is therefore essential for building computational models of liver function and regeneration.

At the organ level, liver microvasculature can be visualized using micro-CT. Corrosion casting obtained by resin perfusion, in combination with micro-CT, has been used to create accurate 3D models of liver vasculature at the organ level under homeostasis, allowing quantification of features such as the hepatic artery, portal vein and hepatic venous trees up to 13 generations (Debbaut et al., 2014). Micro-CT, in combination with the use of phase contrast agents, has been employed to reconstruct progressive revascularization in the regenerating liver (Xie et al., 2016). Results from this study indicated that vascular hepatic growth patterns during regeneration cannot be explained by their hypothesis of isotropic expansion. At smaller length scales, confocal microscopy is commonly used for visualization and characterization of liver vasculature. Additionally, intravital imaging has been utilized for studying blood flow through liver sinusoids (Cabrera and Frevert, 2012). Recent computational modelling of the hepatic circulatory system from the work of Torres Rojas et al. (2021) considered the hepatic artery, portal vein, and hepatic vein as dendritic networks, and each lobule as a component of a porous medium. The model accurately captured the changes in blood pressure and flow rates throughout the hepatic vasculature observed following resection of the liver.

Previous efforts have utilized fluid dynamics to model blood flow within liver lobules (White et al., 2016). However, the high degree of inhomogeneity in liver vascularization requires simplifications to ensure tractability. Microscopic regions of homogeneity, usually lobules or sinusoids, are therefore considered in modeling blood flow (Bonfiglio et al., 2010; Park et al., 2010). In a recent study, a 3D model of the mouse sinusoidal network was generated and visualized following perfusion of the left ventricle and subsequent confocal imaging of the sinusoidal area (Ishikawa et al., 2021). Blood flow and liver volume measurements were taken prior to and at two time points (24 and 120 h) following PHx to study the sinusoidal network dynamics during the initiation and termination phases of liver regeneration. The authors found that mechanical homeostasis, including gravity, shear stress, osmotic pressure, and tension is regulated by cytokine networks. The dynamic changes in mechanical stress and tension in the liver and the signaling processes induced by these changes in the sinusoidal cells occur prior to growth factor production after PHx and together control the initiation and termination of liver regeneration.

Predicative 3D models of bile flow dynamics at various length scales have been developed utilizing immunofluorescence staining and reconstruction techniques (Meyer et al., 2017; Segovia-Miranda et al., 2019). Meyer et al. (2017) generated a spatially resolved model of human liver tissue at different stages of non-alcoholic fatty liver disease (NAFLD). Their findings show that there exists immense topological defects in the bile canalicular network, correlated with NAFLD progression. The multiscale modeling work of Segovia-Miranda et al. (2019) revealed spatial heterogeneities of biliary geometry leading to gradients of bile velocity and pressure in the liver lobule.

## 7 DISCUSSION

Computational modeling is an integral and increasingly important part of systems and predictive medicine. Multiscale phenomena, like liver function under systemic signals and liver regeneration, are inherently complex and require computational approaches to identify points of vulnerability. Describing liver patho-physiology using multiscale modeling approaches has gathered widespread interest (Holzhütter et al., 2012). Transcending spatial and temporal scales requires judicious approximations to make the multi-scale modeling of liver function and regeneration a tractable pursuit. Such approximations could lead to conceptualization of the modeling process at different levels of abstraction. Multiscale models of liver regeneration have been developed without considering explicit histological detail as described in Furchtgott et al. (2009) and Cook et al. (2015). Both models were conceptualized as lumped ODE-based models and considered extracellular matrix and organ-wide cytokine levels as parameters that mediate intercellular communication. Using their lumped multiscale model, Cook et al. (2015) were able to predict the molecular states of hepatic stellate cells that may contribute towards aberrant liver regeneration in rats fed a chronic alcohol diet.

Explicit consideration of simplified lobular histology allows for inclusion of features such as functional zonation into computational models. At the lobular scale, ammonia detoxification has been modeled during liver regeneration by Schliess et al. (2014). This model captures subcellular processes, functional zonation, lobule-level transport, and spatial dynamics of tissue regeneration over periods spanning a few days. Such lobular scale models can be integrated into pharmacokinetic (PBPK) models as seen in Schenk et al. (2017). This opens up the possibility of studying the relationship between extent of tissue damage induced by chemical or surgical intervention and loss of metabolic capacity of the liver. One hypothesis states that the loss of metabolizing tissue is proportional to the loss of metabolic capacity of the organ. Alternatively, the surviving tissue may undergo an adaptive response to compensate for the loss of tissue (compensated loss). A third option is that the loss of metabolic capacity is even higher compared to the fraction of lost metabolizing tissue (aggravated loss). In the case of CCl4 induced acute liver damage, an aggravated loss was observed (Schenk et al., 2017). This type of modeling is required to connect, in a formalized way, key events, such as hepatocyte death, to adverse outcomes, such as metabolic deficiency and liver failure (Leist et al., 2017). Other models have seen integration of whole organism scale, cellular scale and subcellular scale modeling for acetaminophen clearance (Sluka et al., 2016). Including organ histology in models of liver regeneration can help analyze the complex interplay between increased systemic metabolic demands and the resulting spatially localized modes of cytotoxicity, leading to a model capable of predicting regenerative outcomes.

**FIGURE 5 |:**
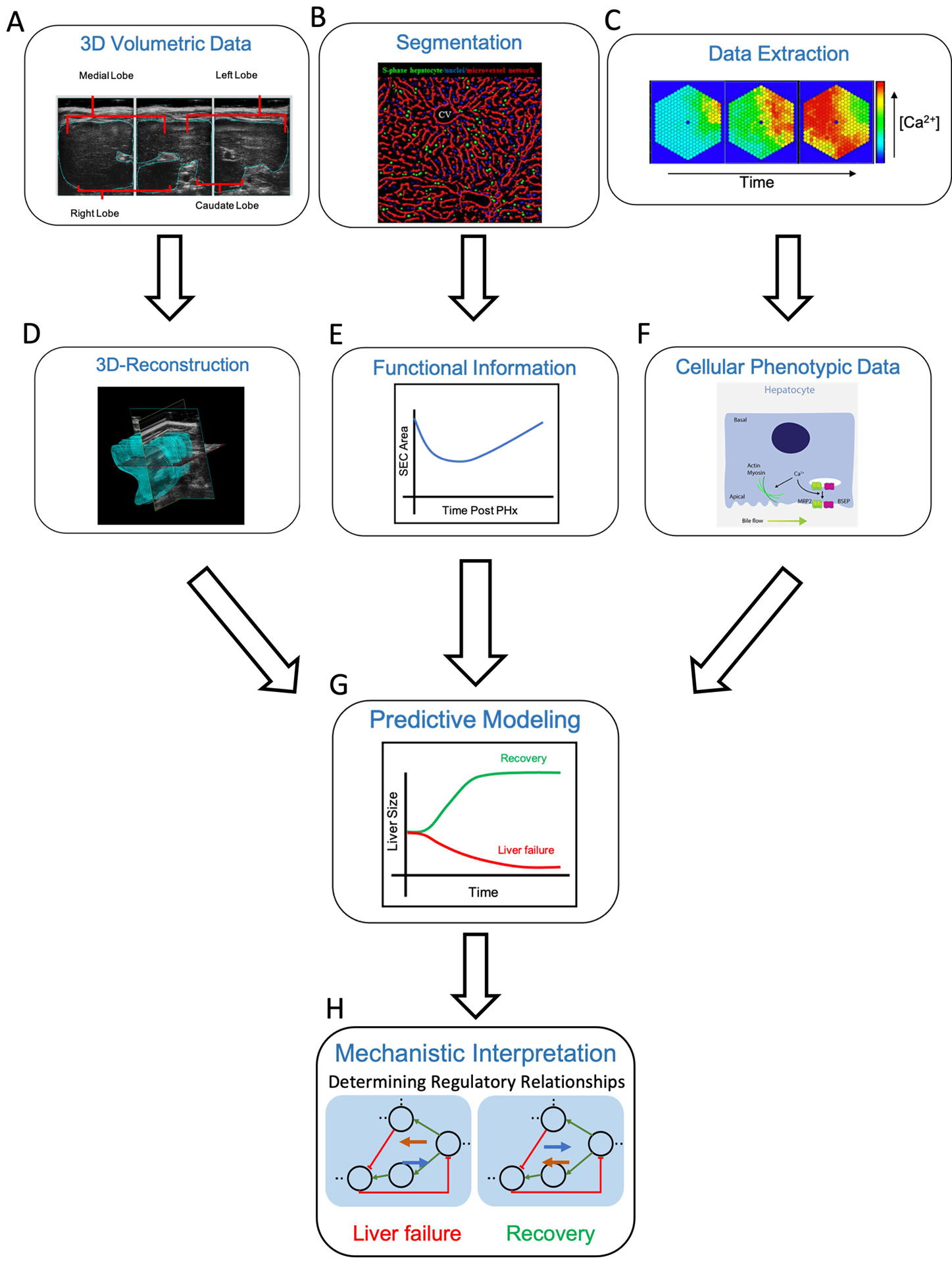
Multiscale modeling approach from “seeing” to “simulating” in the liver. **(A)** Obtaining 3D volumetric data using techniques such as ultrasound (shown), MRI or CT can be used to record changes in liver size, as in the case of liver regeneration. The liver can then be computationally reconstructed **(D)** by stacking the individual volumetric images. **(B)** Segmentation of the hepatic lobule into respective structures, in this case, S-phase hepatocytes (green), S-phase negative nuclei (blue) and the microvessel network (red), allows for extraction of functional information **(E)** such sinusoidal endothelial cell (SEC) area following liver resection by PHx. **(C)** Lobular and cellular-level molecular data can be extracted (i.e., the concentration of cytosolic Ca2^+^ over time) to identify cellular phenotypic changes **(F)** An increase in hepatic calcium levels promotes actin-myosin interactions and bile secretion, through activation of MRP2 and BSEP. Integrating 3D-reconstructed volumetric images **(D)**, functional information from segmentation images **(E)**, and cellular phenotypic data from extracted molecular data **(F)** allows for predicative modeling of liver regeneration **(G)**. A computational model of liver regeneration is then able to provide insights into the mechanistic details and regulatory relationships differentiating situations of liver failure and recovery **(H)** Definitions: BSEP = bile salt export pump, MRP2 = multidrug resistance-associated protein 2. Sources: Maeno et al., 2005; Hammad et al., 2014; Oliva-Vilarnau et al., 2018.

Altogether, with the combined power of imaging and subsequent multiscale modeling of the liver during regeneration, predictions can be made regarding the success or failure of the organ in regenerating to its full functional potential (Figure 5). Specifically, beginning with 3D volumetric data, such as ultrasound, MRI, or CT, one can identify anatomical changes such as organ size following PHx (Figure 5A). Individual images obtained from the 3D volumetric studies can then be stacked for the purpose of 3D reconstruction (Figure 5D). In parallel, imaging at the level of the hepatic lobule, followed by segmentation of known cellular structures can provide information about liver functionality during the regenerative process (Figures 5B,E). Finally, molecular data at lobule and cellular-level resolution can be extracted to identify phenotypic changes throughout the regenerative course (Figures 5C,F). All three paths can be integrated during the predictive multiscale model-building process such that the model is equipped with both functional and anatomical details of liver regeneration (Figure 5G). The model can then be utilized to identify regulatory relationships guiding the liver to regenerative success (i.e., recovery) or failure (Figure 5H).

A recent study showed key progress in demonstrating the potential for combining multiple imaging modalities from the same patient-derived liver tissue sample (Kong et al., 2021). Using multiple imaging methods including light sheet microscopy, Kong et al. (2021) viewed the hepatic architecture from the organ scale down to subcellular resolution. Specifically, the hepatobiliary system was visualized and mapped in 3D using a single patient’s liver biopsy sample, allowing for identification of fibrotic regions extending from the portal field to the parenchyma. Such studies show promise for obtaining multiscale architectural information from an individual patient’s liver tissue. Complementary high resolution and spatially-resolved histopathological data from the same patient’s liver biopsy sample can provide multimodal molecular, cellular and tissue scale information on the functional state of the liver. This data can then be utilized to tune individualized patient models of liver function, disease and repair dynamics.

While *in vivo* imaging provides powerful anatomical and functional information, there are some limitations that may be complemented by the *in vitro* cell culture studies. Specifically, *in vivo* imaging may be limited by low spatial resolution, low sensitivity and poor tissue contrast (Lauber et al., 2017). *In vitro* studies, however, allow for a more precise control of the physiochemical environment that can be manipulated as needed (Arango et al., 2013). The *in vitro* methods allow for high throughput screening approaches to evaluate the contribution of multiple network components to the signaling in liver cells. The *in vivo* imaging on the other hand can provide detailed information on the organ and tissue scales. Liver regeneration as a tissue/organ scale process cannot be studied *in vitro*, where the studies would necessarily have to focus on cellular and molecular aspects. Therefore, complementing *in vivo* imaging with *in vitro* cell culture studies provides new integrative opportunities to develop computational models of liver regeneration with high spatial, temporal, and physiochemical resolution.

**FIGURE 6 |:**
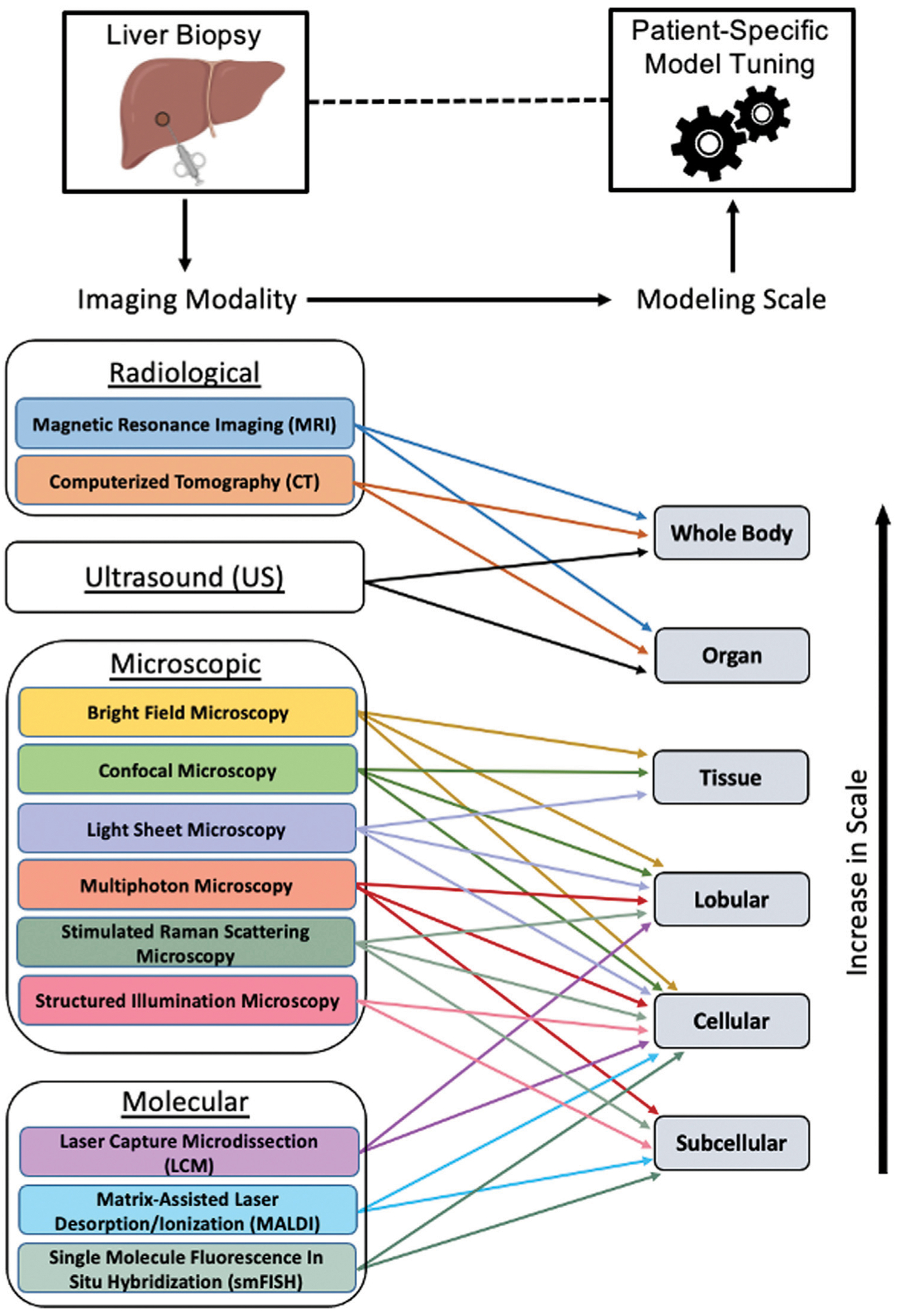
Tuning patient-specific computational models of liver. After obtaining a liver biopsy sample from a patient, various imaging modalities may be utilized for obtaining tissue morphological, histological and functional information used for tuning a multiscale model, thereby aiding in the prognosis and diagnosis of liver conditions. The imaging modalities and the spatial scales at which they can inform a computational model are shown. Figure made using BioRender.

In this review, we focused the discussion on various imaging-based modalities used to enhance the development of multiscale models of the liver (Figure 6). While several of these methods incorporate details of cellular signaling, multi-scale models informed by emerging single cell transcriptomic datasets, may lead to overall model improvements. For instance, in a multiscale model of hepatic calcium signaling, model parameters were constrained such that zonation patterns of calcium signaling components were properly tuned to the referenced single cell RNA-seq data (Verma et al., 2018). In a separate study, Cook et al. (2018) developed a mathematical model of liver regeneration describing the relative contributions of various cell populations in yielding a successful regenerative phenotype. After tuning the model to the acquired single cell gene expression data, four specific hepatic stellate cell transcriptional states were identified and characterized to have a role in liver regeneration. Multiscale models may also benefit from the use of multi-omic (transcriptomic, metabolomic and proteomic) data integration as this provides additional information about the various layers of transcriptional and translational regulation, which aid in building valid, functionally relevant models. Specifically, a recently published review article in the field summarized the importance of integrating single cell expression data into computational models of liver resection (Christ et al., 2021). Despite the relevance of such models, we focus this review on imaging modalities capable of enhancing model simulation and prediction, as this type of modeling can aid clinicians in providing the most optimal care to patients with liver disease.

As the opportunities for personalized medicine continue to evolve, multiscale modeling tuned to patient-specific imaging data can be an important component to assess the disease functional state noninvasively. Lastly, exploiting the latest developments in machine learning and artificial intelligence for imaging and multi-omics data, it is feasible to build faster executable surrogate models that are trained on the intricacies of the imaging data, including the underlying physics and biochemistry. Such models can then predict both the static and dynamic behavior of the tissue during liver disease progression, treatment and post-surgical assessment. In summary, developing hepatic multiscale models using multimodal imaging data can provide a wealth of knowledge supporting translational research and drive potential development of prognostic tools.
